# Emodin alleviates hypertrophic scar formation by suppressing macrophage polarization and inhibiting the Notch and TGF-β pathways in macrophages

**DOI:** 10.1590/1414-431X2021e11184

**Published:** 2021-07-23

**Authors:** Zihuan Xia, Jiancheng Wang, Songlin Yang, Cheng Liu, Shu Qin, Wenbo Li, Yulong Cheng, Huan Hu, Jin Qian, Yi Liu, Chenliang Deng

**Affiliations:** 1 Department of Plastic Surgery, Shanghai Jiao Tong University Affiliated Sixth People's Hospital, Shanghai, China; 2 Department of General Surgery, Rujin Hospital, Shanghai Jiao Tong University School of Medicine, Shanghai, China; 3 Department of Plastic Surgery, Jiangxi Provincial People's Hospital, Nanchang, China

**Keywords:** Hypertrophic scar, Macrophage polarization, Emodin, Notch signaling, TGF-β signaling

## Abstract

Hypertrophic scar (HS) formation is a common complication that develops after skin injury; however, there are few effective and specific therapeutic approaches for HS. Emodin has previously been reported to inhibit mechanical stress-induced HS inflammation. Here, we investigated the molecular mechanisms underlying the inhibitory effects of emodin on HS formation. First, we conducted *in vitro* assays that revealed that emodin inhibited M1 and M2 polarization in rat macrophages. We subsequently established a combined rat model of tail HS and dorsal subcutaneous polyvinyl alcohol (PVA) sponge-induced wounds. Rats were treated with emodin or vehicle (DMEM). Tail scar specimens were harvested at 14, 28, and 42 days post-incision and subjected to H&E staining and Masson's trichrome staining. Histopathological analyses confirmed that emodin attenuated HS formation and fibrosis. Macrophages were separated from wound cells collected from the PVA sponge at 3 and 7 days after implantation. Flow cytometry analysis demonstrated that emodin suppressed *in vivo* macrophage recruitment and polarization at the wound site. Finally, we explored the molecular mechanisms of emodin in modulating macrophage polarization by evaluating the expression levels of selected effectors of the Notch and TGF-β pathways in macrophages isolated from PVA sponges. Western blot and qPCR assays showed that Notch1, Notch4, Hes1, TGF-β, and Smad3 were downregulated in response to emodin treatment. Taken together, our findings suggested that emodin attenuated HS formation and fibrosis by suppressing macrophage polarization, which is associated with the inhibition of the Notch and TGF-β pathways in macrophages.

## Introduction

Hypertrophic scar (HS) is the most common complication after a skin injury, representing a critical skin fibrotic disease that often occurs in response to abnormal skin repair and ulcerative skin defects resulting from chronic wounds and traumatic injuries (1). HS is characterized by excessive deposition of collagen and accumulation of a substantial amount of abnormally secreted extracellular matrix (ECM) proteins. Compared with normal skin, the HS tissue has thicker collagen fibers, disorganized collagen structures, and significant inflammatory responses (2). To date, the etiology of HS is poorly understood, and few effective therapeutic approaches are clinically applied.

Wound healing is complex, consisting of inflammatory, proliferative, and remodeling phases, and HS forms when the inflammatory phase persists (3). Recent findings have indicated that macrophages regulate scar formation and that reducing the numbers of macrophages in the early repair phases can suppress scar formation (4,5). During the inflammatory and proliferative phases, macrophages secrete various soluble factors to stimulate the differentiation, proliferation, and migration of fibroblasts, keratinocytes, and endothelial cells, resulting in new ECM deposition, re-epithelialization, and neovascularization in the wound. Instead of a homogeneous cell population, macrophages in wound repairs are classified as M1 and M2 phenotypes. During the inflammatory phase of wound healing, approximately 85% of macrophages exhibit pro-inflammatory M1 phenotypes, whereas 15% of macrophages exhibit anti-inflammatory M2 phenotypes (6). Pro-inflammatory mediators promote M1 polarization in macrophages, leading to the production of pro-inflammatory cytokines that are responsible for tissue scarring and fibrosis (6). In the middle phase of wound healing, M2 macrophages play a major role in promoting fibroblast transformation into myofibroblasts, leading to secretion of excessive collagen and other ECM proteins to enhance scar formation and fibrosis (4). Therefore, suppressing macrophage polarization during the early phases of wound healing may reduce HS formation and fibrosis (4,5).

The Notch signaling pathway plays an important role in macrophage development and activation (7) and has been reported to contribute to HS formation by modulating keratinocyte phenotype (8). In macrophages, Notch1 signaling promotes pro-inflammatory polarization through IRF8 and NF-κB transcriptional pathways (9). Moreover, the Notch-mediated macrophage polarization participates in the regulation of phagocytosis and tissue fibrosis (10). Transforming growth factor (TGF-β) is another important factor in the wound-healing process and can be secreted by M2 macrophages. Through regulating cell differentiation, collagen production, and ECM degradation (11), TGF-β initiates and terminates tissue repair. The sustained production of TGF-β contributes to the development of fibrosis (12). There have been reports that suppression of the TGF-β signaling pathway represents an effective strategy for limiting excessive scarring (13). It has also been demonstrated that blocking TGF-β/Smad signaling inhibits the development and formation of HS (14).

Emodin is a major component of the Chinese herb rhubarb and has been broadly applied in treating resolving and non-resolving inflammation (e.g., cancer). The potential therapeutic effects of emodin have been investigated in many diseases, including pancreatitis, asthma, arthritis, atherosclerosis, myocarditis, glomerulonephritis, and Alzheimer's disease (15,16). Previous *in vitro* studies have shown that emodin inhibits fibrotic activities in rat kidney fibroblasts and hepatic stellate cells (17) and inhibits mechanical stress-induced HS inflammation (18). However, the *in vivo* effect of emodin on HS and the underlying molecular mechanisms remain unknown. A recent study has demonstrated that emodin suppresses the excessive response of macrophages to both M1 and M2 stimuli and therefore restores macrophage homeostasis in various pathologies (19). In breast cancer, emodin attenuates tumor growth by inhibiting macrophage infiltration and M2 polarization (20). Emodin also suppresses TGF-β1 production in breast cancer cells and macrophages. It, therefore, attenuates TGF-β1 or macrophage-induced metastasis of breast cancer cells (21). However, whether emodin regulates macrophage polarization in HS formation remains unclear.

In this study, we hypothesized that emodin might attenuate HS formation and fibrosis by suppressing macrophage polarization. To test our hypothesis, we explored the regulation of emodin in macrophage polarization in cultured macrophages and a combined rat model of tail HS and dorsal subcutaneous polyvinyl alcohol (PVA) sponge implantation-induced wound. We also investigated the involvement of the Notch and TGF-β pathways in the regulation of emodin in macrophage polarization during HS formation and wound healing. Our results suggest that emodin is a promising therapeutic agent for HS treatment.

## Material and Methods

### Animals

Sprague-Dawley rats (male, 8-12 weeks old, weighing 250-300 g) were provided by the Experimental Animal Center of Shanghai Jiao Tong University Affiliated Sixth People's Hospital (China). Rats were maintained in the animal facility at 23-26°C and in a 12-h light/dark cycle with free access to standard food and clean water. This study was approved by the Animal Ethics Management Committee of Shanghai Jiao Tong University. All experimental procedures were carried out in accordance with the guidelines of the Animal Care and Use Committee of Shanghai Jiao Tong University Affiliated Sixth People's Hospital.

### Peritoneal macrophage isolation and culture

Rats were administered 3 mL of 4% thioglycolate solution via intraperitoneal injection. The macrophages were enriched from peritoneal cavity lavage with phosphate-buffered saline (PBS) and then resuspended in Dulbecco's modified Eagle medium (DMEM; Thermo Fisher Scientific, USA) supplemented with 10% fetal bovine serum (FBS; Sigma-Aldrich, USA). After culturing at an atmosphere of 5% CO_2_ at 37°C for 2 h, non-adherent cells were removed by washing with PBS, and adherent macrophages were further cultured in serum-free DMEM. The dose of emodin for the *in vitro* experiment was established based on the previously described protocol by Iwanowycz et al. (19). To induce polarization, macrophages were stimulated with DMEM containing lipopolysaccharide (LPS; 100 ng/mL; Sigma-Aldrich) and interferon-gamma (IFNγ; 20 ng/mL; BioAbChem Inc., USA) or interleukin 4 (IL-4; 10 ng/mL; BioAbChem Inc.) in the presence or absence of emodin (50 µg/mL; Sigma-Aldrich) for 24 h. The stock solution of emodin (10 mg/mL) was prepared by dissolving emodin in dimethyl sulfoxide and diluted in DMEM as needed.

### Cell proliferation assay

The proliferation of macrophages was determined using a cell counting kit 8 (CCK-8; Dojindo, Japan) as per the manufacturer's protocol. Briefly, macrophages were seeded onto a 96-well plate at a density of 2×10^3^ cells per well in 100 μL medium and cultured for 24 h. After replacing the medium with fresh medium supplemented with emodin at 0, 25, 50, or 75 µg/mL, cells were cultured for an additional 24 h. The CCK-8 reagent was added to the culture medium, and cells were incubated at 37°C for an additional 1 h. The absorbance was measured at 450 nm using a microplate reader (SpectraMax M5; Molecular Devices, USA). Each experiment was repeated in triplicate.

### Tail HS and dorsal subcutaneous PVA sponge implantation-induced wound rat model

Rats were randomly divided into the Control group and the Emodin group (n=15/group). Rats in the Emodin group were intraperitoneally administered emodin at 10 mg/kg body weight (Sigma-Aldrich) once daily, as established by Liu (18). In contrast, those in the Control group received the same volumes of DMEM, intraperitoneally once daily.

A rat tail HS model was established as previously described (22). Briefly, the skin of the tail was disinfected with iodophor (Shanghai Borong Bioscience & Technology Co., Ltd., China). The wound region was marked with methylene blue (Sigma-Aldrich). The panniculus carnosus was removed to create a 7×7 mm^2^ wound using scalpels and iris scissors. The wound was covered with a dry sterile gauze after hemostasis. Mechanical stretching was started immediately after the operation. The abdominal side of the rat tail was fixed with customized stainless-steel rings (2-cm in diameter). The tail was bent following the curvature of the steel ring (Supplementary Figure S1). Three rats from each group were sacrificed at 14, 28, and 42 days post-incision to obtain the scar samples (n=3 rats for each group; n=9 samples for each group) for histopathological examination.

A dorsal PVA sponge implantation wound model was established in the same rat as previously described (23). Briefly, the rat was anesthetized with pentobarbital (Alfa Chemistry, USA; 50 mg/kg body weight) via intraperitoneal injection. A total of 3 sterile PVA sponges (4 mm; PVA Unlimited, USA) were implanted in the dorsal skin of the rat. Rats were sacrificed at 3 and 7 days after PVA implantation for wound cell isolation and macrophage separation.

### Wound cell isolation and macrophage separation

Rats were sacrificed 3 and 7 days after PVA implantation. Wound cells were collected as previously described (24). Briefly, the PVA sponges were compressed 20 times in 1 mL sterile PBS using blunt forceps, followed by centrifugation at 300 *g* for 5 min at 4°C to separate the cells and supernatant.

Macrophages were separated from the wound cells as previously described (25). First, Fc receptors were blocked with anti-FcRγIII/II (1 µg/1×10^6^ cells, #sc-166711; Santa Cruz Biotechnology, USA) for 15 min at 4°C. Then, cells were incubated with phycoerythrin (PE)-conjugated antibodies against CD2 (0.1 µg/1×10^6^ cells, #MA5-17488; Thermo Fisher Scientific) and CD5 (0.5 µg/1×10^6^ cells, #MA5-17407; Thermo Fisher Scientific). Next, the cells were washed, and depletion of neutrophils and lymphocytes was achieved by incubating the cells with Anti-PE MicroBeads (#130-048-801, Miltenyi Biotec, Germany 2 mL/1×10^9^ cells) and subsequent negative selection using a MACS system (Miltenyi Biotec), following the manufacturers' instructions. The purity of macrophage suspensions varied from 90 to 95%, as determined by CD68 immune staining (Supplementary Figure S2).

### Histopathological examination

After fixing with 10% formaldehyde, specimens were embedded in paraffin and subjected to staining with hematoxylin and eosin (H&E) or Masson's trichrome reagent following standard methods. Briefly, scar samples from tails of the rats sacrificed at 14, 28, and 42 days post-incision were embedded in paraffin and cut into 5-μm-thick tissue sections. The sections were de-waxed in xylene, rehydrated through graded alcohol, washed in distilled water, and stained with H&E. The collagen fibers in the scar samples were stained with Masson's trichrome.

We randomly selected 5 high-power (HP) field count observation indicators for each slice. Histopathological changes and the blue-stained collagen fibers were observed using optical microscopy (Panoramic DESK, 3D HISTECH Ltd., Hungary) at magnification 100× or 400× regarding scar elevation index (SEI), collagen structure, and inflammation. The ratio of scar thickness to that of the adjacent normal skin was denoted as SEI as follows (26): 0, SEI 0-1; 1, SEI 1-2; 2, SEI 2-3; 3, SEI ≥ 3. Collagen structure was scored as follows: 0, well organized with no whorl; 1, disorganized with no whorl; 2, disorganized with one whorl per HP field; 3, disorganized with ≥2 whorls per HP field. Inflammation was scored as follows: 0, no monocytes; 1, 2-5 monocytes or mast cells per HP field; 2, 5-10 monocytes or mast cells per HP field; 3, >10 monocytes or mast cells per HP field. Samples were scored by two experienced pathologists independently in a double-blinded manner. The average subscores were then summed up to obtain the final histopathological scores.

### Flow cytometry

For surface antigens staining, Fc receptors of target cells were blocked with anti-FcRγIII/II antibodies (1 µg/1×10^6^ cells; #sc-166711; Santa Cruz Biotechnology). Then, the cells were washed twice with staining buffer (PBS containing 2% FBS). To distinguish monocytes, macrophages, and granulocytes, cultured cells or those harvested from PVA sponges were stained with fluorochrome-conjugated antibodies against the following cell surface markers: CD2 (0.1 µg/1×10^6^ cells, #MA5-17488; Thermo Fisher Scientific), CD5 (0.5 µg/1×10^6^ cells, #MA5-17407; Thermo Fisher Scientific), CD68 (1:100, #ab31630; Abcam, UK ), CD86 (1:100, #ab213044; Abcam), and Arg1 (1:50, #93668S; Cell Signaling Technology, USA). Dead cells were excluded by propidium iodide (PI; Thermo Fisher Scientific) staining. Data were acquired using a flow cytometer (FACS Canto II; BD Biosciences, USA) and analyzed by FlowJo software (BD Biosciences).

### Western blotting

Macrophages were homogenized in radioimmunoprecipitation lysis buffer (Thermo Scientific). Protein samples were obtained by centrifugation for 10 min at 14,000 *g* at 4°C. Samples were separated by 10% sodium dodecyl sulfate-polyacrylamide gel electrophoresis and transferred onto polyvinylidene difluoride membranes by the wet blotting procedure (100 V for 2 h at 4°C). Membranes were blocked with blocking buffer (5% skimmed milk in PBS containing 0.05% Tween-20) and incubated at 4°C overnight with the anti-Notch1 (1:1000, #4380; Cell Signaling Technology), anti-Notch4 (1:1000, #ab184742; Abcam), anti-Hes1 (1:1000, #11988S; Cell Signaling Technology), anti-TGF-β (1:1000, #ab31013; Abcam), anti-Smad3 (1:1000, #9523S; Cell Signaling Technology), or anti-GAPDH (1:1000, #2118S; Cell Signaling Technology) primary antibody. GAPDH was used as an internal control. After incubation of the membrane with an IRDye 800 anti-rabbit or IRDye 680 anti-mouse secondary antibody (LI-COR, USA) for 1 h at room temperature, densitometric analysis was performed using an Odyssey infrared imaging system (LI-COR).

### Reverse transcription-quantitative polymerase chain reaction (RT-qPCR)

Total RNA from macrophages was purified using the RNAiso plus reagent (Takara, China) following the manufacturer's instructions. cDNA was synthesized using 500 µg RNA per reaction with PrimeScript RT Master Mix (Takara) according to the manufacturer's instructions. A 7300 Real-Time PCR system (Applied Biosystems, USA) was used for amplification. The qPCR program was set as follows: enzyme activation at 95°C for 30 s, followed by 40 cycles of 95°C for 5 s and 58°C for 31 s. The primers used for qPCR are summarized in Table 1.

**Table 1:** Primers used in quantitative reverse transcription polymerase chain reaction.

Gene	Forward	Reverse
Notch1	5′-TCGTGCTCCTGTTCTTTGTG-3′	5′-TTCTCTCCGCTTCTTCTTGC-3′
Notch4	5′-GGATGAATGTCGGAGTGACC-3′	5′-GGCTACACAAGGGAACCTCA-3′
Hes1	5′-GTGGGTCCTAACGCAGTGTC-3′	5′-GTCAGAAGAGAGAGGTGGGCTA-3′
TGF-β	5′-ATTCCTGGCGTTACCTTGG-3′	5′-AGCCCTGTATTCCGTTCTCT-3′
Smad3	5′-GGTAAAGGATTGCCACCAAA-3′	5′-GAACAGCCAGGAAAGGGACT-3′
GAPDH	5′-CGACCACTTTGTCAAGCTCA-3′	5′-AGGGGTCTACATGGCAACTG-3′

### Statistical analysis

Statistical analysis was performed using Prism 7.0 (GraphPad Software, USA). Data are reported as means±SD except when otherwise specified and are representative of at least three independent experiments. Differences among multiple groups were evaluated by one-way analysis of variance followed by Newman-Keuls *post hoc* test. Group pairs were compared using a two-tailed Student's *t*-test. A P value <0.05 was considered statistically significant.

## Results

### Emodin downregulated M1 and M2 polarization rates of macrophages *in vitro*

**Figure 1:**
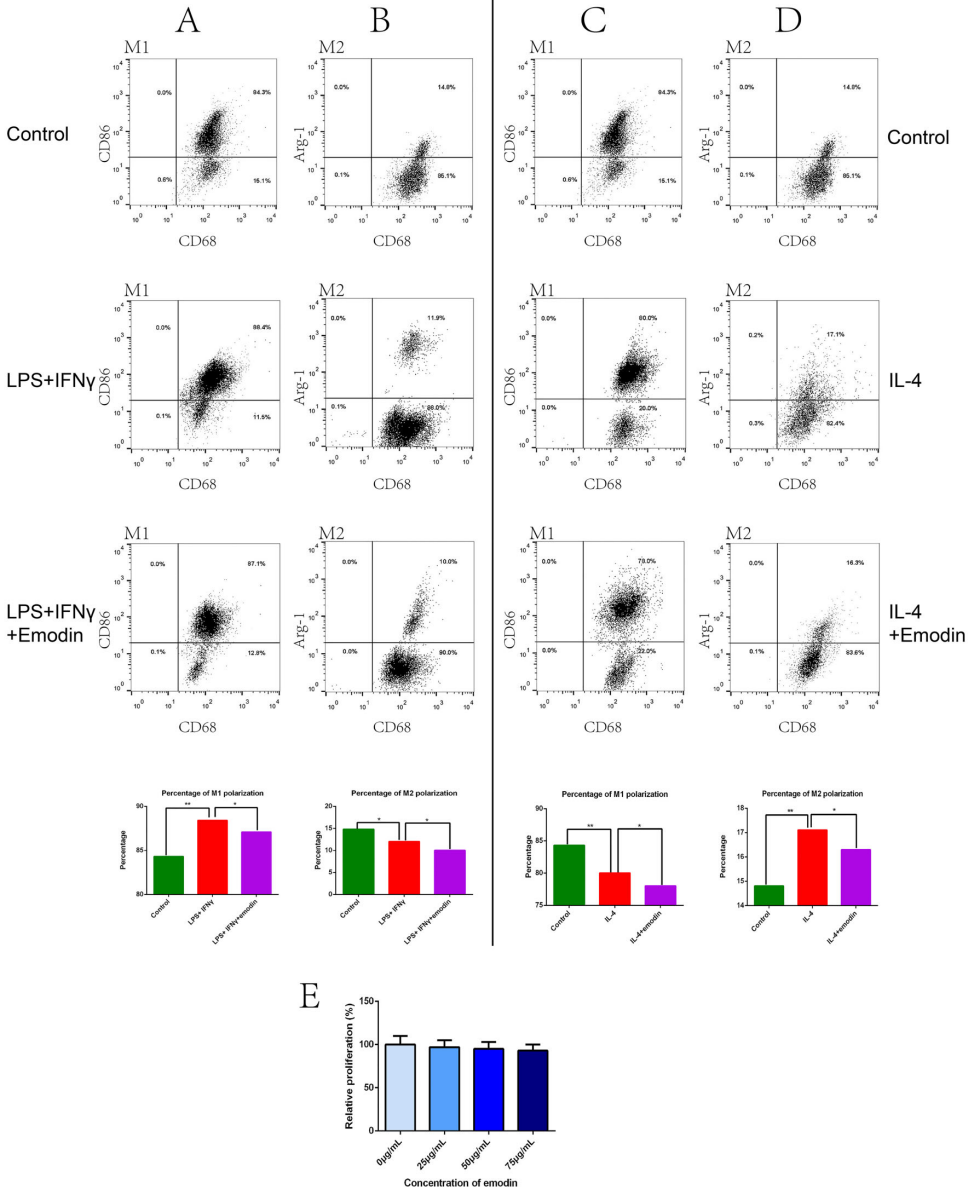
Emodin modulated M1 and M2 polarization of macrophages *in vitro*. **A**-**D**, Macrophages isolated from male Sprague-Dawley rats were treated with M1-inducing factors (100 ng/mL lipopolysaccharide (LPS) and 20 ng/mL interferon-gamma (IFNγ)) or M2-inducing factor (10 ng/mL interleukin 4 (IL-4)) in the presence or absence of emodin (50 µg/mL) for 24 h. Untreated cells were used as a negative control. The immune phenotypes of cells were assessed by flow cytometry. Representative flow profiles denoting the percentages of CD86^+^ M1 macrophages (**A** and **C**) or Arg-1^+^ M2 macrophages (**B** and **D**) among CD68^+^ total macrophages are shown (upper panels). The percentages of M1 or M2 macrophages are summarized (lower panels). **E**, The relative proliferation of rat macrophages treated with emodin at the indicated concentrations was measured with CCK-8 assay. Data are reported as means±SD for n=3 for each group. *P<0.05; **P<0.01, between the indicated groups (ANOVA followed by Newman-Keuls *post hoc* test).

First, we evaluated the potential effects of emodin on *in vitro* polarization of macrophages cultured in the presence of M1 polarization factors LPS and IFNγ or M2 polarization factor IL-4. Compared with the Control group, the LPS+IFNγ group had a significantly increased M1 polarization rate (the percentage of CD86^+^ cells) and M2 polarization rate (the percentage of Arg-1^+^ cells) and emodin treatment partially but significantly reversed the polarization factor-induced increase in the M1 or M2 polarization rate of macrophages (P<0.05; Figure 1A). On the other hand, compared with the Control group, the LPS+IFNγ group had a lower M2 polarization rate, probably because more macrophages were polarized into the M1 phenotype (Figure 1B).

Similarly, the IL-4 group had a lower M1 polarization rate than the Control group (Figure 1C). Compared with the Control group, the IL-4 group also had a significantly increased M1 polarization rate (the percentage of CD86^+^ cells) and M2 polarization rate (the percentage of Arg-1^+^ cells) and emodin treatment partially but significantly reversed the polarization factor-induced increase in the M1 or M2 polarization rate of macrophages (P<0.05; Figure 1D).

Notably, the LPS+IFNγ+Emodin group or the IL-4+Emodin group had a further decreased M2 polarization rate or M1 polarization rate, respectively. This suggested that emodin inhibited M2 macrophage polarization under M1-inducing conditions and inhibited M1 macrophage polarization under M2-inducing conditions. Viability showed that emodin ranging from 0-75 µg/mL did not change the proliferative ability of macrophages *in vitro* (Figure 1E), suggesting that emodin less than 75 µg/mL was safe for macrophages. Taken together, emodin inhibited both M1 and M2 polarization of macrophages *in vitro*.

### Emodin administration alleviated HS formation in rats

**Figure 2:**
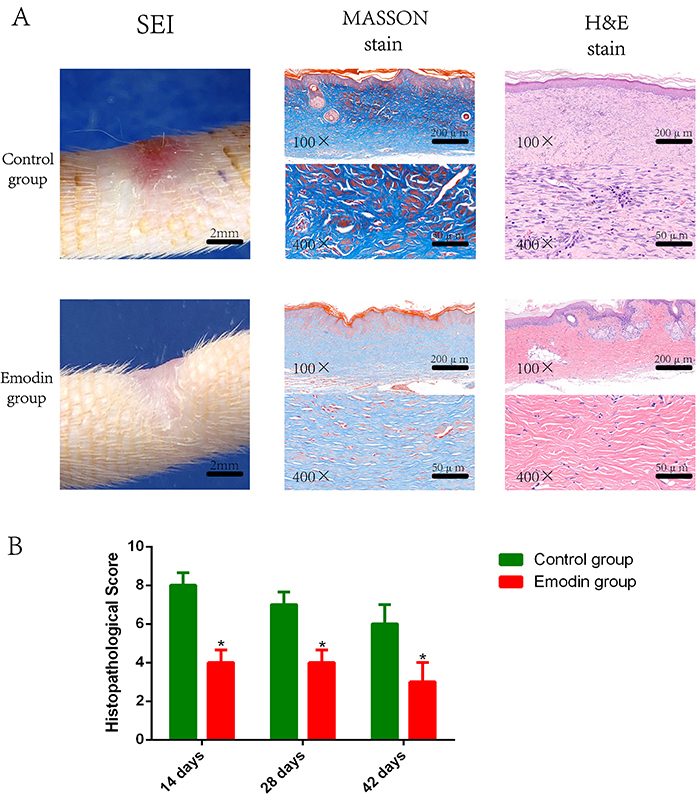
Histopathological changes induced by emodin treatment in a rat model with tail hypertrophic scar. **A** and **B**, After modeling of the tail hypertrophic scar in rats, the animals were treated with vehicle or emodin. The scar samples at 14, 28, and 42 days after modeling were assessed and scored for scar elevation index (SEI), collagen structure, and inflammation. **A**, Representative images show the results of SEI (left), Masson's trichrome staining (fibrosis; middle), and hematoxylin and eosin (H&E; inflammation) staining (right) at 28 days after modeling. **B**, Histopathological scores at the indicated times (sums of all three subscores of inflammation, fibrosis, and SEI) in the Control and Emodin groups are summarized. Scores were obtained from experienced pathologists in a double-blinded manner. Data are reported as means±SD for n=9 for each group. *P<0.05 *vs* the Control group (two-tailed *t*-test). Scale bars, 2 mm in the SEI images, 200 μm in the 100× field, and 50 μm in the 400× field in the micrographs of Masson's or H&E stain.

To assess the effects of emodin on HS formation *in vivo*, we established a rat tail HS model. We treated the rats with vehicle or emodin daily. To capture the process of scar formation in the model, we collected the specimens from rats euthanized at 14, 28, and 42 days post-incision, and SEI, collagen structure, and inflammation in the specimens were scored using the criteria as described in the methods. Each score from emodin-treated rats of these parameters was lower, by at least one grade (the interval between two adjacent score levels) than that of the Control group. As shown in Figure 2A, scar thickness was reduced after emodin administration, and no collagen whorls were observed in the specimens with markedly reduced inflammatory cells. Treatment with emodin resulted in significantly decreased histopathological scores than the Control group on days 14, 28, and 42 post-incision (Figure 2B). These results indicated that emodin administration suppressed inflammation and HS formation in rats.

### Emodin inhibited M1 and M2 polarization of macrophages in PVA sponges *in vivo*

**Figure 3:**
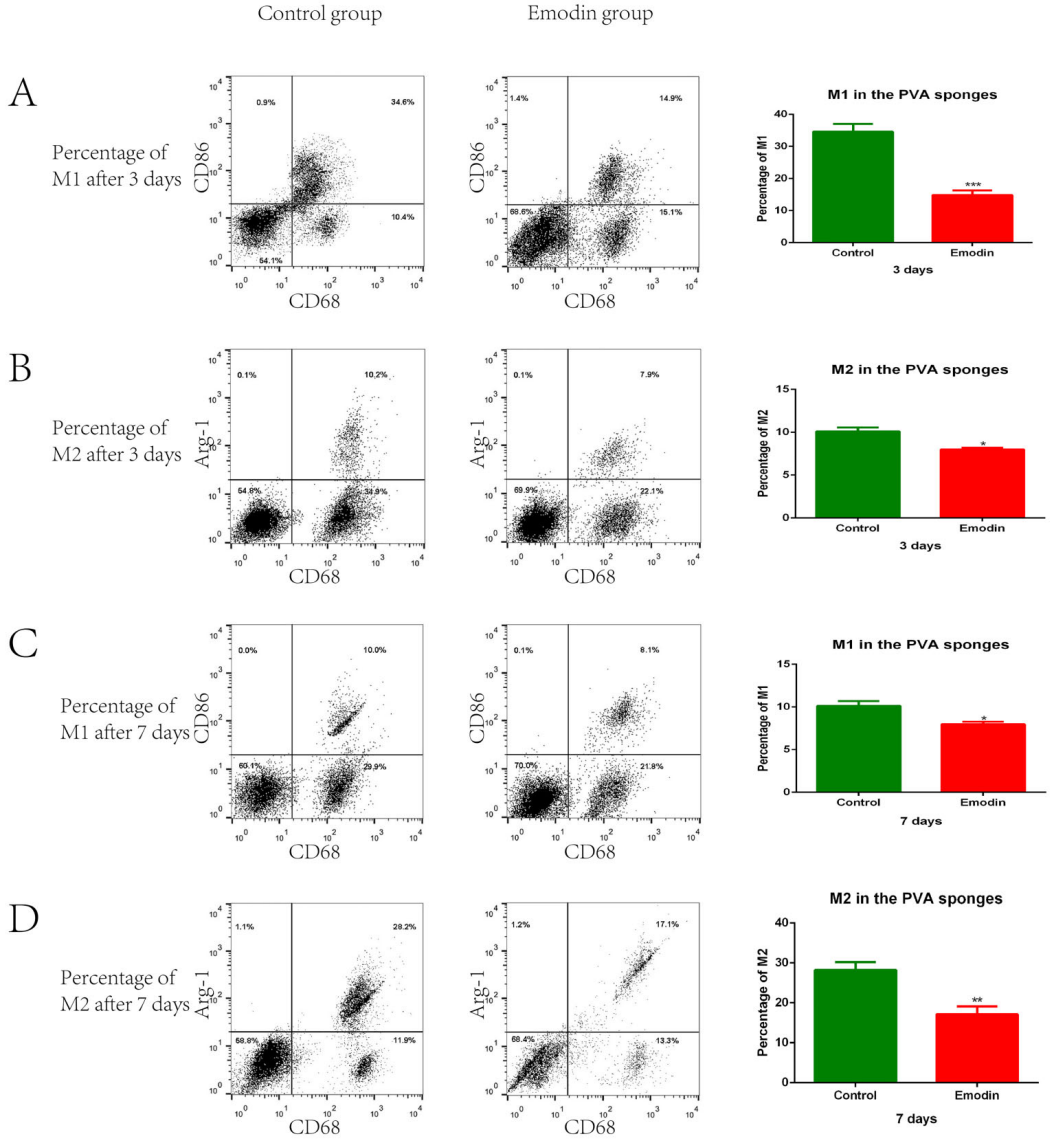
Emodin modulated *in vivo* M1 and M2 polarization of macrophages in implanted polyvinyl alcohol (PVA) sponges. **A**-**D**, The implanted PVA sponges were removed and assessed for M1 (**A** and **C**) and M2 (**B** and **D**) populations at 3 days (**A** and **B**) and 7 days (**C** and **D**) after implantation. The percentages of M1 and M2 macrophages and total macrophages in sponge exudates were determined by flow cytometry using the indicated markers. Representative flow profiles are shown (left), and the percentages of M1/M2 populations are summarized (right). Data are reported as means±SE for n=3 for each group. *P,0.05, **P<0.01, ***P<0.001 *vs* the Control group (two-tailed *t*-test).

To characterize phenotypes of macrophages at the early- and mid-stages of wound healing with or without emodin administration, we implanted PVA sponges in the dorsal skin of the same rat with tail HS. Flow cytometry revealed significantly reduced percentages of CD68^+^ macrophages and weakened polarization of M1 macrophages (CD86^+^-cells) in emodin-treated rats at 3 days (early-stage) after wound formation (P<0.01, Figure 3A). Compared with the Control group, the Emodin group also had a significantly lower M2 polarization rate (Figure 3B). At 7 days after wound formation (the mid-stage), the percentage of M2 macrophages (Arg-1^+^ cells) was significantly reduced in the Emodin-treated group, compared with the Control group (P<0.01; Figure 3C). Compared with the Control group, the Emodin group also had a significantly lower M1 polarization rate (Figure 3D). These findings indicated that emodin inhibited M1 and M2 polarization in PVA sponge-induced wounds in rats.

### Emodin downregulated TGF-β expression in PVA exudates

**Figure 4:**
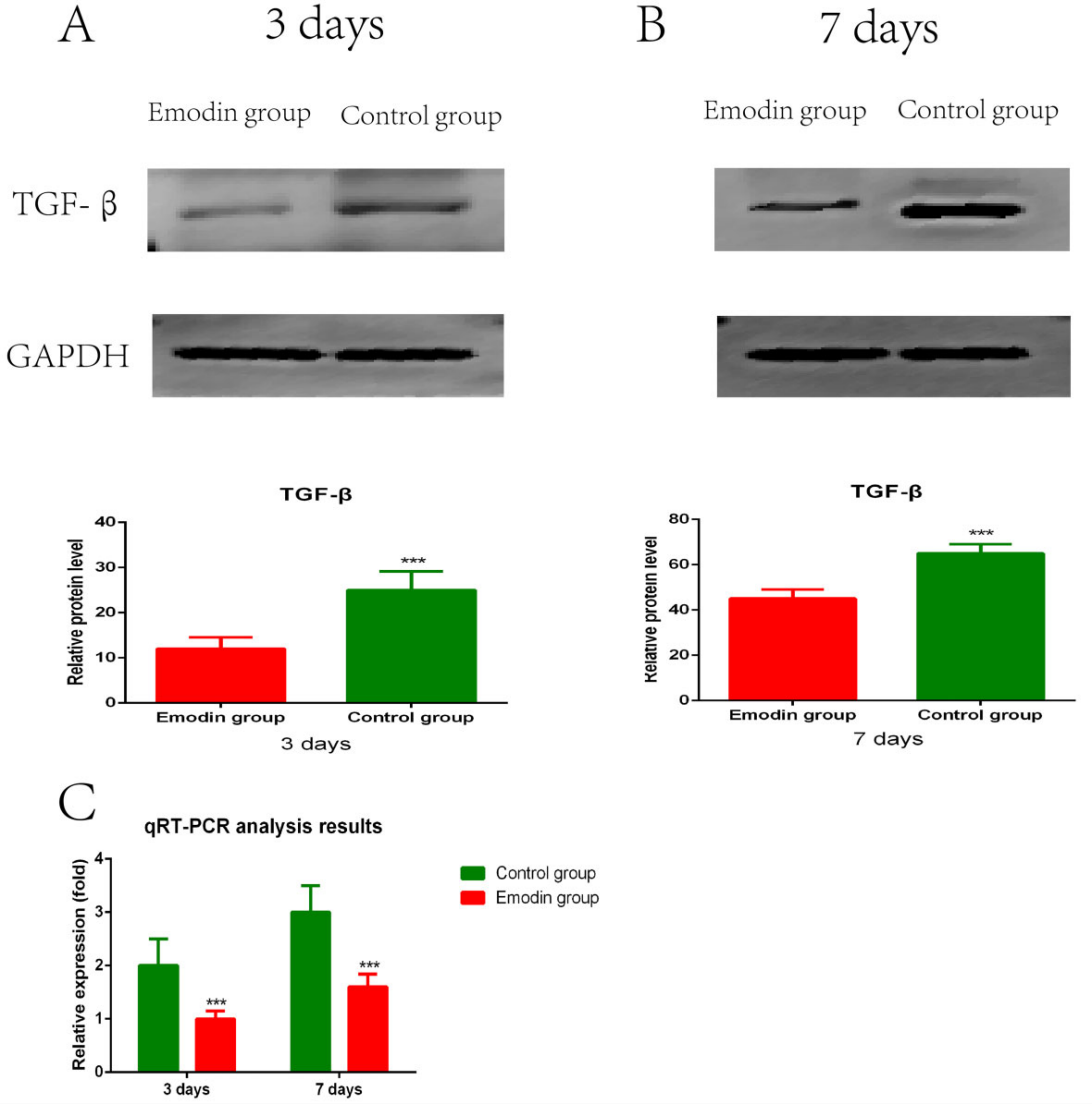
Emodin suppressed transforming growth factor (TGF)-β expression in implanted polyvinyl alcohol (PVA) sponges at 3 and 7 days post-injection. **A**-**C**, PVA sponges were removed at 3 and 7 days after implantation. Macrophages were isolated from PVA sponge exudates and assessed for TGF-β expression at the protein (**A** and **B**) and mRNA (**C**) levels by western blot and qPCR, respectively. GAPDH was used as a reference in these assays. Representative images of western blot bands are shown, and relative expression of TGF-β is summarized (**A** and **B**). Data are reported as means±SE for n=3 for each group. ***P<0.001 *vs* the Control group (two-tailed *t*-test).

To further investigate the molecular mechanisms underlying emodin-suppressed HS formation and fibrosis, we determined TGF-β expression in PVA exudates (15). Western blot analysis and qPCR showed that emodin treatment significantly downregulated protein and mRNA expression of TGF-β in PVA exudates compared with vehicle treatment (Figure 4A-C) on days 3 and 7 after wound formation. On day 3 and 7, the differences between the Emodin and Control groups were statistically significant (both P<0.001), indicating that TGF-β expression levels were sustainably reduced by emodin at least on day 7. Collectively, these results suggested that TGF-β downregulation was involved in emodin-suppressed HS formation and fibrosis in rats.

### Emodin inhibited the Notch and TGF-β signaling pathways in macrophages from PVA sponge-induced wound in rats

**Figure 5:**
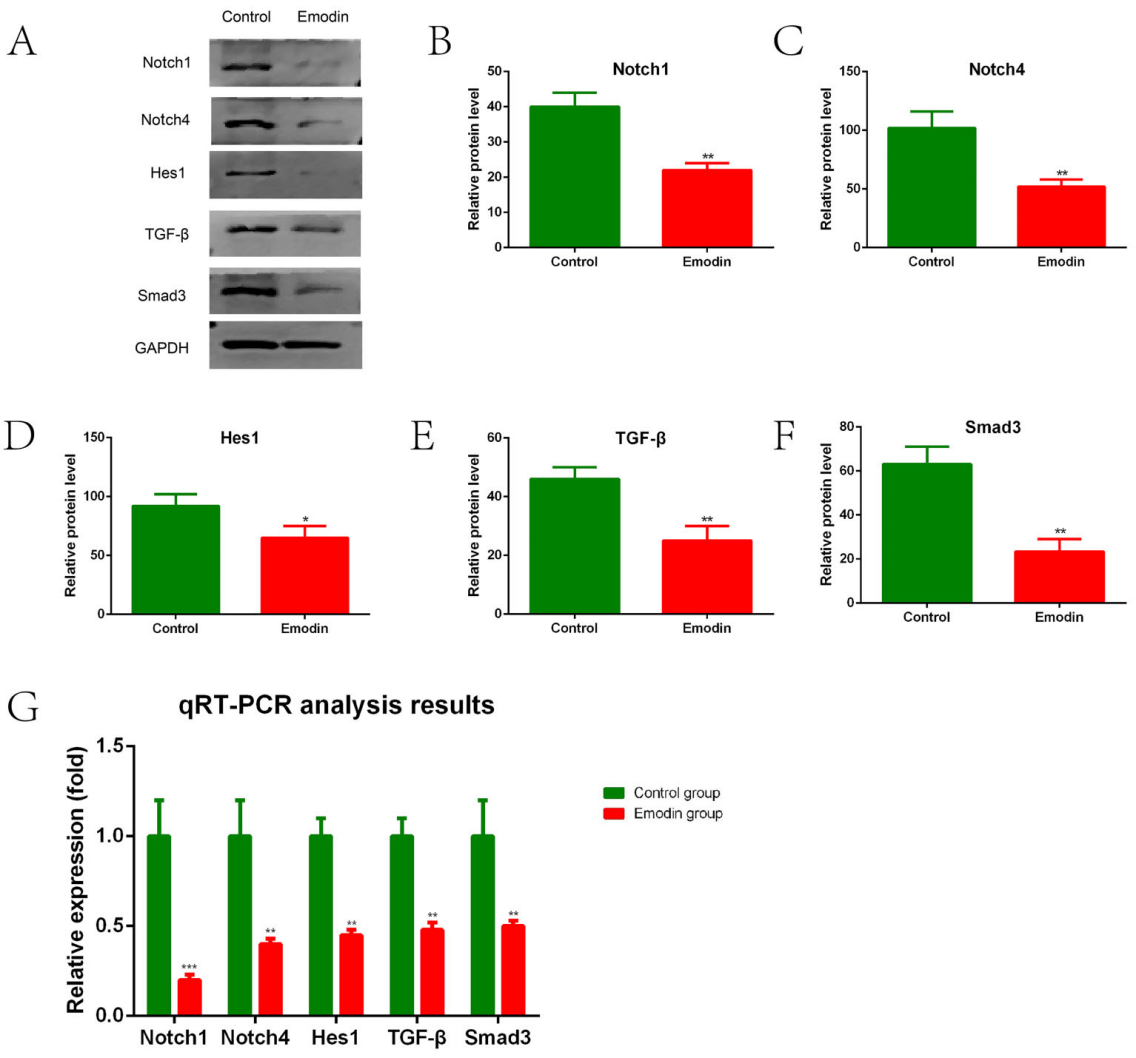
Emodin suppressed the transforming growth factor (TGF)-β and Notch signaling pathways in macrophages in implanted polyvinyl alcohol (PVA) sponges. **A**-**G**, Macrophages were isolated from the implanted PVA sponges 7 days after implantation. The expression of TGF-β and Notch pathway effectors in macrophages at the protein (**A**-**F**) and mRNA (**G**) levels were assessed by western blot and qPCR, respectively. Representative images of western blot bands are shown (**A**), and relative expressions of the indicated molecules (**B**-**F**) are summarized. GAPDH was used as a reference in these assays. Data are reported as means±SE for n=3 for each group. *P<0.05, **P<0.01, ***P<0.001 *vs* the Control group (two-tailed *t*-test).

Since Notch and TGF-β signaling converges in HS formation regulation (8,27), we further evaluated the expression of key molecules in these signaling pathways in macrophages isolated from PVA exudates. The macrophages from emodin-treated rats had significantly decreased protein levels of Notch1, Notch4, and Hes1 compared with those from the Control group (Figure 5A; Notch1, P<0.01, Figure 5B; Notch4, P<0.01, Figure 5C; Hes1, P<0.05, Figure 5D). Similar results were observed in TGF-β (Figure 5E) and Smad3 (Figure 5F) expression. Consistent results were observed in qPCR (Figure 5G). These data suggested that modulation of macrophage polarization by emodin was associated with the suppression of Notch and TGF-β signaling.

## Discussion

Effective and specific therapeutic approaches are lacking in HS treatment due to the poor understanding of HS etiology and the limited source of candidate medicines (1). Macrophages coordinate and sustain the wound-healing process, playing crucial roles in HS formation and fibrosis. In this study, we explored the molecular mechanisms of emodin in alleviating HS *in vitro* and *in vivo*. First, we demonstrated that emodin modulated M1/M2 macrophage polarization *in vitro*. In addition, emodin suppressed scar formation and fibrosis in a rat tail HS model, involving the suppression of M1 and M2 macrophage polarization, TGF-β expression, and the TGF-β and Notch pathways in macrophages. To the best of our knowledge, this is the first study providing *in vivo* evidence that emodin suppresses HS formation and fibrosis, possibly by suppressing macrophage polarization and TGF-β and Notch signaling.

Given the limited access to clinical specimens, multiple animal models have been developed for experimental studies of HS pathogenesis, with few achieving satisfactory results (28,29). In this study, we selected a novel HS rat model as previously described (22), in combination with PVA sponge dorsal implantation in rats. In this combined model, PVA sponge-induced early inflammatory cell infiltration and subsequent ECM deposition and neovascularization resulted in a collagenous scar fully encapsulating the sponge, mimicking the cellular events that occur during skin injury (28). The PVA sponge dorsal implantation model is a classic wound healing model that facilitates effective isolation and analysis of infiltrating macrophages in the wound sites during the healing process (23). The rat tail scar model has advantages, such as simple operation and good simulation effects, that were confirmed in our preliminary experiments. In addition, the rat tail model exhibits minor wound contraction and has biological features similar to both normotrophic and hypertrophic scars in humans, serving as a good model for studying skin wound healing and scar formation (22). However, it was difficult to extract sufficient cells infiltrated into the tail scars. We failed to implant the PVA sponge under the tail skin due to technical difficulties. Therefore, we took advantage of the benefits of these two models by inducing tail scar and implanting dorsal PVA sponge in the same rat, considering that the macrophages collected from the PVA sponges can represent the baseline state of macrophages during HS formation. In fact, we have conducted experiments in two models separately, and similar results were observed (data not shown), further supporting our findings. However, the macrophages in PVA sponge exudates might not fully represent the macrophages functioning during HS formation, which is a limitation of this study.

The scar formation mechanism is generally complicated with a relatively unknown cause. Several studies have shown that the number and subtype ratio of macrophages in the process of wound healing affects the formation of scars (23). In the early stage of wound healing, the macrophages are mainly M1, described as the pro-inflammatory type. In the early stage of healing, these promote the degree of inflammation around the wound, and the scar formation is related to the severity of the inflammation (23). On the other hand, in the middle and late stages of wound healing, the macrophages are mainly M2 type described as the repair type. They promote the formation and deposition of ECM protein and collagen. Excessive collagen deposition increases the formation of hypertrophic scars, and the number and proportion of M1 and M2 macrophages are constantly changing during the wound healing process (23). Thus, the inhibition of both the M1 and the M2 polarization can eventually inhibit the formation of scars from both the early inflammatory response of the wound and the excessive repair of the wound in the middle and late stages.

In this study, we demonstrated that emodin suppressed both M1 and M2 macrophage polarization *in vitro* and in the combined model. Emodin also effectively inhibited HS formation and fibrosis in rats, consistent with other studies (18,19). Histopathological analysis revealed that emodin modulated macrophage polarization, an effect that was more evident at 3 days post-injury in M1 macrophages and 7 days in M2 cells. This is consistent with the trend of M1/M2 macrophage populations under physiological conditions (5,30). Furthermore, scars in emodin-treated rats were thinner than those in vehicle-treated rats, along with reduced ECM protein deposition. This indicated that reduced M1/M2 macrophages might gradually inhibit hypertrophic scar formation and fibrosis. A possible mechanism is that emodin inhibited M1 macrophage polarization to suppress the inflammatory response at the early stage of wound healing, leading to reduced hypertrophic scar formation. We also showed that emodin inhibited macrophage recruitment and extrusion in PVA sponges, leading to reductions in M1 and M2 macrophages and total macrophages in the wound sites. Previous studies have suggested that fewer macrophages in the early stages of wound healing are beneficial for reducing scar formation (5,31). Reduced numbers of macrophages in the wound may be another mechanism by which emodin inhibits scar formation. The decreased population of M2 macrophages also led to reduced repair in the middle and late phases of wound healing along with lower TGF-β secretion, thereby suppressing scar fibrosis. Taken together, these findings suggest that emodin suppresses the inflammatory response and scar formation by inhibiting macrophage recruitment and polarization.

M2 macrophages infiltrated into the wound site produce TGF-β (18), a multifunctional growth factor that regulates cell proliferation, migration, and differentiation and ECM production and immune modulation (32). High levels of TGF-β expand the fibroblast population and promote fibroblast differentiation into myofibroblasts, increasing collagen production and strengthening the wound (32). However, excess collagen induces fibrosis. To further assess TGF-β expression and changes in M2 macrophages, immunoblot and qPCR assays were performed to determine TGF-β levels in PVA sponge exudates. We found that TGF-β levels in the wound exudate were significantly decreased at 3 and 7 days post-incision in emodin-treated rats compared with those in control samples, suggesting emodin suppresses TGF-β expression at the wound sites. In line with our results, previous investigations have reported that emodin inhibits the TGF-β/SMAD pathway (33) and that upregulated TGF-β expression results in delayed wound healing and promotes HS formation (34). These findings collectively indicate that low TGF-β levels in the wound can reduce HS formation and fibrosis.

To further elucidate the molecular mechanisms of emodin, we assessed the expression levels of the effectors of the TGF-β and Notch pathways that are involved in wound healing (35). Differential regulation of Notch receptors and autonomous activation of Notch signaling in M1-polarized macrophages are associated with the activation of the Toll-like receptors in macrophages (36). In the present study, the Notch pathway was activated in wound-derived macrophages after injury and seemed to be more activated at 7 days post-injury. These results are consistent with recent studies indicating that Notch-related genes remain at low levels within epidermal cells while being activated after injury (25,37). At 7 days post-injury, M2 macrophages usually constitute the main macrophage population, assisting wound healing during the proliferation phase (30,35). Therefore, our results indicated that M2 macrophages might be activated by the Notch pathway and release TGF-β to improve the skin repair process and promote scar formation. Several studies have assessed emodin's effect on the Notch pathway with divergent conclusions. For example, one study has indicated that emodin reduces TGF-β1-induced upregulation of Notch-1 expression in the nucleus and cytoplasm (38). In addition, it has been demonstrated that emodin suppresses Notch 1-3 and DLL4 production (39). However, other studies have shown that emodin upregulates Notch1 and downregulates Jagged1, VEGF, and bFGF at the mRNA and protein levels (40). In this study, emodin suppressed Notch 1, Notch 4, and Hes1 expression and inhibited the Notch pathway in macrophages isolated from PVA sponges, suggesting that downregulation of Notch signaling contributed to suppressed wound macrophage polarization in emodin-treated rats.

The following are the limitations of this study: i) the two phenotypes of M1 and M2 are still unsolved scientific problems; they are not absolute separate phenotypes; ii) the application of single-cell nuclear sequencing to subdivide macrophage subtypes further was not carried out in this research; iii) the macrophages in PVA sponge exudates in our study might not fully represent the macrophages functioning during HS formation; and iv) an added benefit could have been to show other components of the ECM involved in fibrosis in histological sections such as the expression of cell proliferation marker proliferating cell nuclear antigen (PCNA) and α-smooth muscle actin (α-SMA) or collagen subtypes.

In conclusion, this study supported previous findings regarding the inhibitory effect of emodin on scar formation. It revealed that emodin-mediated suppression of M1/M2 macrophage polarization contributed to this effect. Importantly, emodin modulated macrophage M1 polarization to reduce the inflammatory response at the early stage of wound healing and regulated macrophage M2 polarization to suppress TGF-β expression at the middle and late stages. Moreover, emodin attenuated HS formation and fibrosis by suppressing the TGF-β and Notch pathways in macrophages at wound sites. Our work provides valuable insights into the development of emodin-based therapeutics for HS treatment.

### Data availability

The datasets used and/or analyzed during the current study are available from the corresponding author on request.

## Supplementary Material

Click here to view [pdf].
